# Use of CBCT in Orthodontics: A Scoping Review

**DOI:** 10.3390/jcm13226941

**Published:** 2024-11-18

**Authors:** Alessandro Polizzi, Sara Serra, Rosalia Leonardi

**Affiliations:** Department of General Surgery and Surgical-Medical Specialties, School of Dentistry, University of Catania, 95124 Catania, Italy

**Keywords:** orthodontics, review, cone beam computed tomography, digital dentistry

## Abstract

**Objectives**: The present scoping review aims to provide a panoramic view of the current state of knowledge, highlighting the strengths, limitations, and future directions, on the use of CBCT in orthodontic practice. **Methods**: This study followed the Preferred Reporting Items for Systematic Reviews and Meta-Analyses extension for Scoping Reviews (PRISMA-ScR) guidelines to identify eligible studies from the following databases: PubMed, Scopus, and Web of Science. The research question was formulated as follows: “What is the scientific evidence concerning the preferential use of 3D CBCT over 2D radiography in orthodontics”? **Results**: Through database searching, 521 records were identified, and ultimately, 37 studies that compared 3D CBCT with 2D conventional radiography were included. Of these, 16 articles regarded the use of CBCT for cephalometric analysis, 5 papers analyzed the evaluation of root resorption, 10 studies evaluated the diagnostic accuracy of root angulation and determining tooth position, and the remaining 6 articles were conducted for miscellaneous applications: determining the size of the nasopharyngeal airway (n = 2), miniscrew positioning (n = 1), estimating cervical vertebrae maturity (n = 1), and evaluating the correctness of the root location when placing digital indirect brackets (n = 1). **Conclusions**: The choice between 3D CBCT or CBCT-generated radiography and conventional 2D radiography in orthodontics involves careful consideration of the specific clinical context, the complexity of the case, and the balance between the diagnostic advantages and the associated limitations. **Future Directions**: Future studies with a prospective design and standardized imaging protocols are encouraged to facilitate the development of a consensus on the best practices.

## 1. Introduction

In the dynamic field of orthodontics, the relentless pursuit of innovative diagnostic and treatment modalities has significantly enhanced the precision and efficacy of orthodontic interventions. One such groundbreaking technology that has revolutionized the landscape of orthodontic imaging is Cone Beam Computed Tomography (CBCT). As orthodontic practice continues to evolve, the integration of CBCT has emerged as a powerful tool, offering three-dimensional (3D) imaging capabilities that surpass traditional two-dimensional (2D) radiography [1,2,3,4].

One of the most significant drawbacks of 2D radiography is its inability to provide a complete three-dimensional (3D) representation of the craniofacial complex. This limitation can result in difficulties in accurately assessing the spatial relationships and dimensions of anatomical structures, potentially leading to less precise diagnoses and treatment plans [5,6]. In 2D radiography, overlaps between anatomical structures are common, making it challenging to discern the precise position and orientation of the teeth, roots, and surrounding tissues. Superimposition can obscure important details, hindering an orthodontist’s ability to visualize individual structures clearly. Moreover, 2D radiography is susceptible to distortion and magnification errors, which can compromise the accuracy of measurements. Variations in the projection angles and the distances from the X-ray source can lead to inaccuracies in the representation of dental and skeletal structures, affecting the reliability of cephalometric analyses [7,8]. Two-dimensional radiography provides two-dimensional images that may not accurately represent the true density and quality of bone. This limitation is crucial in orthodontics, where an understanding of bone density is essential for assessing the feasibility and stability of orthodontic movements [9,10,11]. Airway assessment is increasingly recognized as an integral aspect of orthodontic diagnosis and treatment planning [12]. However, 2D radiography has limitations in accurately evaluating the size and morphology of the airways, potentially overlooking issues related to airway obstruction or constriction [13,14].

In this regard, 3D Cone Beam Computed Tomography (CBCT) has been proposed as an advanced diagnostic method for assessing various conditions in orthodontics [15]. For example, CBCT was assessed for evaluating changes in alveolar bone and root resorption after orthodontic treatment [16], for cephalometric analysis [17], and for upper airway analysis in patients with sleep breathing disorders [18].

However, while CBCT offers significant advantages in orthodontic imaging, it is not without its limitations, including those related to image artifacts (streaking, scatter, and metal artifacts), limited soft tissue contrast, and interpretation expertise. Understanding these limitations is crucial for clinicians to make informed decisions regarding the appropriate use of CBCT in orthodontic practice [19,20,21].

This scoping review [22] delves into the scientific rationale behind using CBCT in orthodontics, exploring its potential impact on diagnosis, treatment planning, and follow-up. Through a comprehensive analysis of the existing literature, this article aims to provide a panoramic view of the current state of knowledge, highlighting the strengths, limitations, and future directions, of CBCT in orthodontic practice, shedding light on its role in cephalometric analysis, diagnosis, and treatment evaluation. By critically examining the evidence base, this scoping review attempts to contribute to a deeper understanding of the scientific underpinnings that underscore the judicious incorporation of CBCT into orthodontics, fostering a more informed and evidence-based approach to patient care.

## 2. Materials and Methods

### 2.1. Research Strategy

This scoping review followed the PRISMA-ScR guidelines [23]. Using a combination of MeSH terms and free text words pooled through Boolean operators (‘AND’ and ‘OR’) in the PubMed, Scopus, and Web of Science databases, a search strategy without a timeline set was carried out on 4 December 2023 to find all articles related to the use, indications, and limitations of 3D CBCT in orthodontics. The research question was formulated as follows: “What is the scientific evidence concerning the preferential use of 3D CBCT over 2D radiography in orthodontic diagnosis, treatment, and follow-up”?

### 2.2. Inclusion Criteria and Data Extraction

In this scoping review, exact inclusion and exclusion criteria were used to guide the selection of the articles (Table 1). Specifically, publications written in English were only accepted if they provided a comparative analysis of 3D CBCT and 2D radiography in the field of orthodontics. The following publications were taken into account for the study design: cross-sectional studies, cohort studies, case–control studies, randomized and non-randomized controlled clinical trials, and retrospective investigations. Conversely, all articles that had no bearing on the use of 3D CBCT over 2D radiography in orthodontics, were not fully textually available in English, and included certain study designs (such as opinion pieces, theses, conference reports, case reports, case series, and any type of review articles) were not included.

The methods for selecting the articles involved two separate authors, who used EndNote^TM^ 20 desktop version (Clarivate, 1500 Spring Garden Street, Fourth Floor, Philadelphia, PA 19130, USA, October 2020) for the screening and selection of relevant studies [24]. The first screening of the articles was applied to the titles and abstracts after duplicates were eliminated. Every article that met the inclusion criteria had its full-text version examined in more detail. If there was any controversy following the comparison of the final findings, a third researcher was consulted. The article and the year of publication, the aim, the sample, the study’s design, the indication, and the results were all extracted from the papers included. 

## 3. Results

### 3.1. Paper Selection

**Figure 1:**
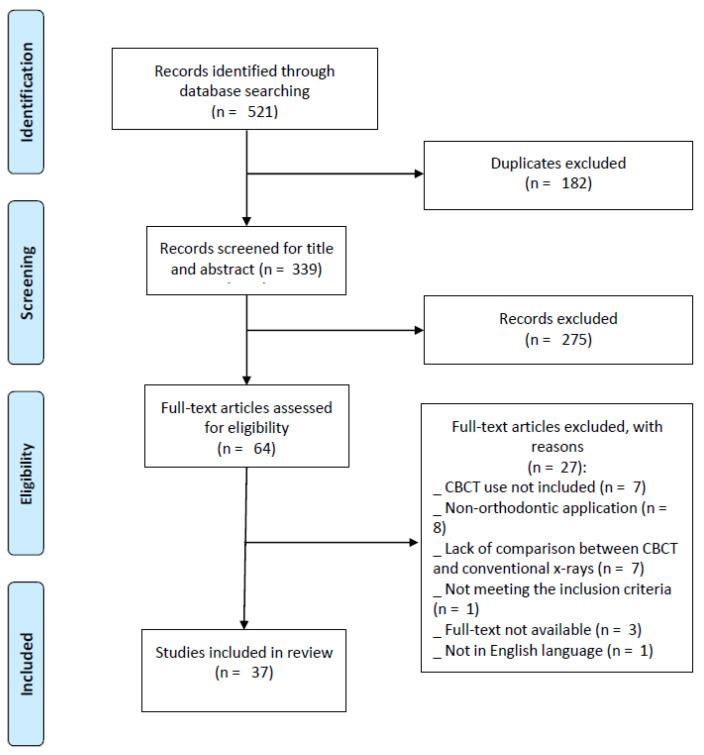
PRISMA-ScR flow diagram of the study identification.

The following research strategy was adopted: Orthodontic* AND (CBCT OR 3D OR “cone-beam computed tomography”) AND (2D OR radiograph* OR orthopantomography OR teleradiograph*) AND (cephalometr* OR superimposition OR impacted OR distortion OR measurement OR tooth OR canine OR eruption OR “bone density” OR airway) AND (comparison OR versus OR vs.). Through database searching, 521 records were identified (Figure 1): PubMed (n = 210), Scopus (n = 220), and Web of Science (n = 91). After the removal of 182 duplicates, 339 records were screened for their titles and abstracts, leading to the exclusion of 273 non-eligible papers. A total of 66 full-text articles were assessed for eligibility, and 27 studies were excluded for the following reasons: CBCT use not being included (n = 7), non-orthodontic applications (n = 8), a lack of comparison between CBCT and conventional radiography (n = 7), not meeting the inclusion criteria (n = 1), the full text not being available (n = 3), and not being in English language (n = 1). Finally, 37 studies were included in the present scoping review.

### 3.2. CBCT for Cephalometric Analysis

**Figure 2:**
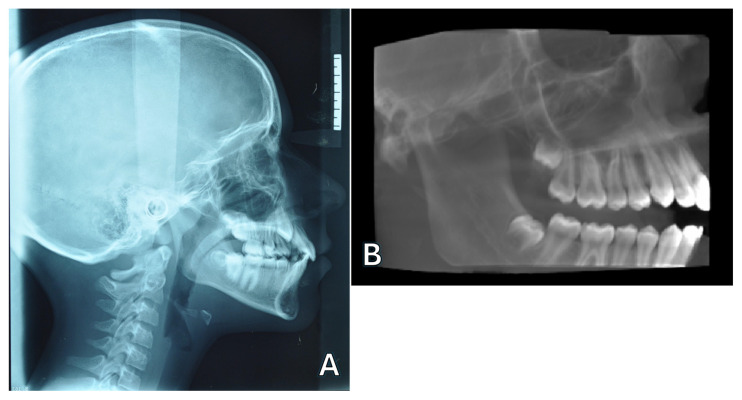
(**A**) 2D conventional lateral cephalogram and (**B**) lateral cephalogram obtained from 3D CBCT.

In Table 2, 16 articles regarding the use of CBCT for cephalometric analysis have been included and summarized. Regarding the study design, seven papers were cross-sectional [25,26,27,28,29,30,31], five articles were observational studies in dry skulls [32,33,34,35,36], and the remaining four were retrospective [37,38,39,40]. These articles compared 2D lateral cephalograms with 2D reconstructions derived from CBCT or directly with the 3D CBCT volume (Figure 2). Within the first typology, the various studies focused on comparing the accuracy of angular and linear measurements between CBCT-generated cephalograms and conventional 2D lateral cephalograms [25,29,34,36,37,40] or on comparing their accuracy for landmark recognition [26,30]. Regarding direct comparisons of 3D CBCT vs. 2D conventional cephalograms, three articles [27,35,39] evaluated the intra-observer and inter-observer reliability in landmark recognition, whereas the remaining seven studies compared 3D vs. 2D cephalometric measurements [28,31,32,33,35,38,39].

### 3.3. CBCT in the Evaluation of Root Resorption

**Figure 3:**
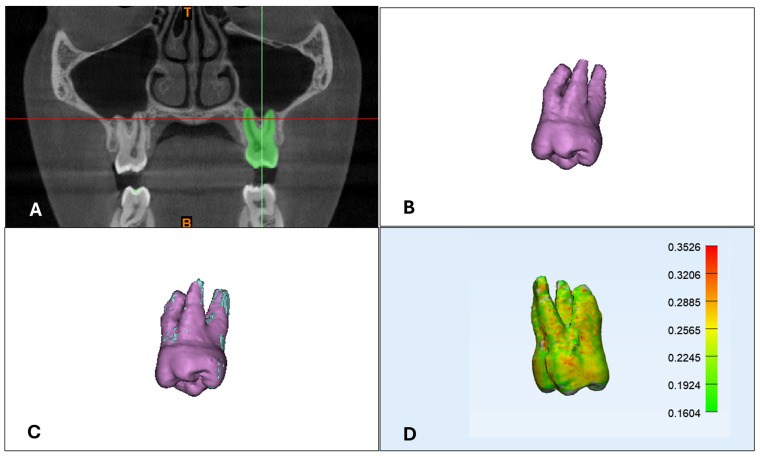
Three-dimensional evaluation of first upper molar root resorption: (**A**) tooth segmentation from 3D CBCT; (**B**) 3D rendering of first upper molar, (**C**) first upper molar superimposition between baseline and follow-up evaluation; and (**D**) deviation analysis.

In Table 3, five articles regarding the use of CBCT to evaluate root resorption have been included and summarized. Regarding the study design, two papers were retrospective studies [41,42], one was cross-sectional [43] and the remaining two were observational in studies in dry skulls [44] or in vitro studies [45]. The number of subjects included in the in vivo studies ranged from 20 to 27 patients, whereas the other studies were conducted on a skull from a child cadaver and on 19 extracted teeth. Root resorption was evaluated using 3D CBCT (Figure 3) and periapical and panoramic radiography.

### 3.4. CBCT in the Evaluation of Root Angulation and Tooth Position

In Table 4, 10 articles regarding the use of CBCT to evaluate root angulation or tooth position have been included and summarized. Regarding the study design, five studies were retrospective [46,47,48,49,50], two were prospective cohort studies [51,52], two were cross-sectional [53,54], and 1 was cross-over [55]. The number of subjects included ranged from 20 to 118 patients. Four papers evaluated the impact of the use of CBCT on the diagnostic information and the related treatment plan for impacted upper canines compared to 2D conventional radiography [47,51,52,55]. In the remaining articles, the authors focused on the evaluation of mesiodistal root angulation or position and teeth parallelism [46,48,49,50,53,54]. Comparisons were performed between panoramic radiography (PAN) and 3D CBCT [46,47,48,51,52,53,54,55] and/or panoramic images from CBCT (PAN-CBCT) [49,50,53,54].

### 3.5. CBCT for Miscellaneous Applications

**Figure 4:**
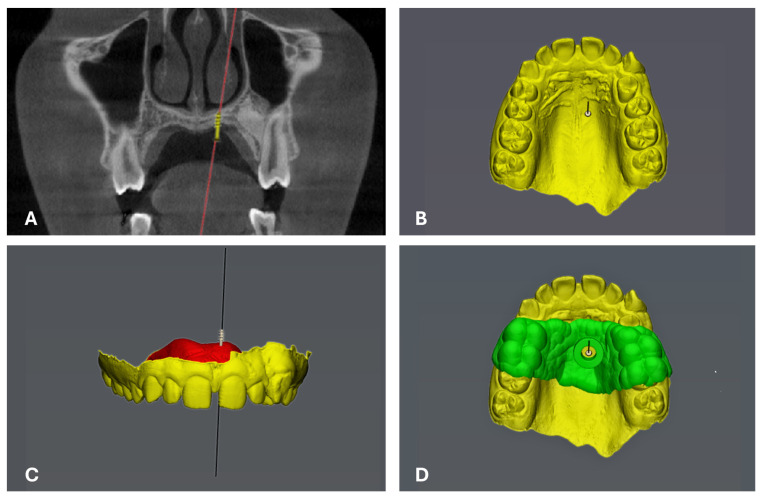
Use of 3D CBCT for palatal TAD insertion: (**A**) coronal view of final TAD position; (**B**) occlusal view of the 3D model; (**C**) frontal view of the 3D model; and (**D**) surgical guide for TAD insertion.

In Table 5, six articles regarding the use of CBCT for miscellaneous applications have been included and summarized. Two articles were retrospective studies [56,57], one was a prospective cohort study [58], two were cross-sectional [59,60], and one was an observational study in dry skulls [61]. Regarding the field of CBCT applications and its comparison with 2D conventional radiography, two articles focused on determining the size of the nasopharyngeal airway [58,59], one article evaluated the accuracy of miniscrew positioning [61] (Figure 4), another study estimated the cervical vertebrae maturity (CVM) [56], and one paper estimated the accuracy of transverse jaw–base and dental relationship measurements [60], whereas the last study evaluated the correctness of the root location when placing digital indirect brackets [57].

## 4. Discussion

The comprehensive analysis presented in this scoping review elucidates the use of CBCT in orthodontics, particularly in comparison to conventional 2D radiography, across various crucial dimensions of orthodontic evaluation. In the field of cephalometric analysis, CBCT may emerge as a transformative imaging modality, providing a 3D perspective that surpasses the limitations of traditional 2D radiography [62]. The enhanced spatial visualization offered by CBCT facilitates more precise measurements of craniofacial structures, enabling orthodontists to glean detailed insights into skeletal relationships and facial proportions [63]. Moreover, the exploration of root resorption evaluation reveals CBCT’s superiority in detecting and quantifying root resorption, offering a more comprehensive understanding of the impact of orthodontic forces on tooth structures [64]. The assessment of root angulation and tooth position further underscores CBCT’s advantages, allowing for a meticulous examination of the dental anatomy in three dimensions, thus aiding in more accurate treatment planning and execution [65]. As for the size of the pharyngeal airway, CBCT may emerge as a valuable tool for evaluating the airway dimensions with greater precision than that in 2D radiography, providing crucial information for orthodontic interventions aiming to address airway-related issues [66]. Additionally, the three-dimensional imaging capabilities of CBCT enable orthodontists to meticulously plan and carry out miniscrew placement, contributing to the efficacy and stability of orthodontic treatments [67]. However, these diagnostic and treatment planning advantages must be weighed against the exposure to ionizing radiation involved, which requires compliance with the ALADA “as low as diagnostically acceptable” principle [68]. But it is necessary to implement this concept into clinical practice [69]. At this point, the analysis of the present scoping review aims to elucidate the clinical rationale for the use of CBCT in orthodontics, based on clinical applications according to the data from the literature and ALARA “As Low As Reasonably Achievable” and ALADA guidelines [68,69].

### 4.1. CBCT and Conventional 2D Radiography for Cephalometric Analysis

The utilization of 3D CBCT or CBCT-generated cephalograms in orthodontic cephalometric analysis has garnered significant attention, presenting a myriad of advantages and disadvantages when compared to conventional lateral cephalograms [70]. Proponents of 3D imaging argue that CBCT offers a more comprehensive view of the craniofacial complex, surpassing the limitations of conventional two-dimensional imaging [71]. The advantages of 3D CBCT include the ability to visualize structures in three dimensions, providing detailed insights into skeletal relationships, facial proportions, and anatomical nuances that may be obscured in traditional lateral cephalograms. This enhanced spatial visualization is particularly valuable in assessing complex cases, facilitating more precise treatment planning and execution [72]. However, some older 3D CBCT devices for cephalometric analysis could involve exposure to higher radiation compared to modern conventional cephalograms. This could raise concerns about potential health risks, particularly in pediatric patients or in cases requiring repeated imaging [73]. But recent evidence-based recommendations from the American Dental Association [74] have suggested that most of the newer CBCT systems provide clinical scanning protocols with a lower-dose setting. Moreover, operators can reduce the radiation dose to the patient via using the smallest possible field of view needed for the clinical purpose [74]. Regardless, conventional lateral cephalograms remain a staple in orthodontic diagnosis due to their familiarity and low radiation exposure. However, their limitations in providing a complete three-dimensional representation of craniofacial structures and the potential for overlaps between anatomical features can compromise the precision of cephalometric analysis [75].

In this regard, the studies comparing 2D lateral cephalograms with 2D reconstructions derived from CBCT [25,29,34,36,37,40] converged in reporting that there were no significant differences in the measures between the two imaging approaches and that conventional headfilms can be effectively replaced by cephalograms generated using CBCT. It seems that when measurements are made in cephalometric radiographs derived from CBCT scans, the repeatability (intra-observer reliability) may be higher than that of measurements made in traditional cephalometric radiography [34]. However, in the study by Aksoy S, et al., it was reported that measurements on curved surfaces were not easily reproducible for 3D rendering software (involved in the generation of CBCT-derived cephalograms), and future efforts in this specific field should focus on designing new landmarks and analysis tools in tandem with the advancement of 3D imaging [37]. Regardless, these findings suggest that for orthodontic diagnosis, CBCT scans may therefore typically be obtained without the need for further conventional imaging [29]. However, CBCT exams should only be performed when the 3D data included can enhance the course of therapy [40].

Regarding the comparisons between 2D lateral cephalograms and 3D CBCT cephalometry, most of the studies reported a good level of agreement between the 2D and 3D CBCT measurements [28,31,32,33,38,39]. However, the reorientation technique in 3D CBCT may alter the measures. For example, Jung PK, et al. [38] detected some alterations in the measures, although they were not clinically significant. In contrast, van Vlijmen OJ, et al. [35] reported that a clinically significant disparity between measurements made in traditional cephalometric radiography and 3D models was discovered for a select few parameters. The consequence is that measurements made in 3D models of the same skull may differ dramatically from measurements made in traditional cephalometric radiography. Therefore, the authors concluded that when there are only 2D recordings from the past, the authors advise against using 3D tracings for longitudinal evaluations. These contrasting results may be explained by the different methodologies applied and the study designs. Regardless, all three articles [27,35,39] evaluating the intra-observer and inter-observer reliability in landmark recognition concluded that when compared to 2D conventional cephalograms, CBCT provided generally enhanced the intra-observer and inter-observer reliability for certain landmarks.

### 4.2. CBCT and Conventional 2D Radiography in the Evaluation of Root Resorption

According to the results of the present scoping review, one of the primary advantages of 3D CBCT is its ability to provide a comprehensive, three-dimensional visualization of dental structures. This allows for a more detailed and accurate assessment of root resorption compared to conventional 2D radiography, where overlapping structures may hinder precise identification. CBCT facilitates volumetric analysis of the entire tooth structure, enabling orthodontists to evaluate the extent and location of root resorption more comprehensively. This is particularly beneficial in cases where resorption may occur in less accessible areas, as it provides a complete 3D view of the affected tooth. Moreover, the high spatial resolution of CBCT allows for precise measurements of root resorption craters, aiding in quantifying the severity of resorption. This detailed information is invaluable for treatment planning and monitoring the progression of root resorption over time. In this regard, all the studies [41,42,43,44,45] converged in reporting that panoramic radiography (PAN) is less reliable or not reliable compared to CBCT in diagnosing and measuring root resorption. This may be particularly important when it is necessary to evaluate the location of palatally displaced canines and to identify canine-induced maxillary incisor root resorption [43]. In particular, small resorptions appeared to be more challenging to document and could only be reliably evaluated using CBCT [41]. Two articles [41,45] also reported the results of periapical radiography (PA). Alamadi E, et al. observed that when it came to the evaluation of linear measurements, PAN was very different from CBCT and PA. However, regarding measuring and rating slanted root resorptions, CBCT was the most precise method available. In an in vitro study performed on extracted teeth, Saccomanno S, et al. reported that for the diagnosis of root resorption, PAN was not helpful, whereas PA examination was the most reliable and impartial tool for identifying root resorption. The authors concluded that 2D PA radiography could be a good choice, especially for routine imaging and monitoring, where the comprehensive 3D information provided by CBCT may not be necessary, except in cases where anatomical overlaps do not allow the prognosis of the tooth involved to be established with certainty and, consequently, it may the most appropriate orthodontic therapeutic approach for the individual clinical case.

### 4.3. CBCT and Conventional 2D Radiography in the Evaluation of Root Angulation and Tooth Position

The primary advantage of 3D CBCT lies in its ability to provide a comprehensive, three-dimensional visualization of dental structures. This allows orthodontists to assess root angulation and tooth position with greater accuracy, as the 3D images offer insights into the spatial relationships among the teeth and surrounding structures. Regarding the impact of the use of CBCT on the diagnostic information and related treatment plans for impacted upper canines, most of the related studies [47,51,52,55] reported significant advantages compared to 2D conventional radiography. However, in a prospective cohort study, the researchers concluded that CBCT scans may provide valuable orthodontic treatment planning information that is comparable to that from conventional records. This may be due to the different evaluations and study design compared to those in the other articles. In fact, in instances with more severe maxillary canine impaction, CBCT may be reasonably indicated. More specifically, according to Wriedt S, et al.’s findings, when the canine inclination in PAN exceeds 30°, when root resorption of the neighboring teeth is suspected, and/or when the canine apex is not clearly discernible, implying dilaceration of the canine root, small-volume CBCT may be justified as an addition to routine PAN.

Regarding the evaluation of mesiodistal root angulation or root position and teeth parallelism, most of the authors found that different information may be obtained on tooth position (particularly with regard to the position of the mesiodistal apex and the position of the labio-palatal cusp) from the analyses of PAN vs. CBCT image reconstructions [46,48,49,50,53,54]. However, the observations of the various authors differed from each other. For example, according to Alquareer A, et al. [46], PAN-based clinical decisions regarding root angulation had a comparable statistical reliability to and substantial agreement with CBCT-based clinical decisions. Instead, Bouwens DG, et al. [48] showed variation in the root angulation between the values obtained from PAN and CBCT images. Barakaat AA, et al. [53] observed that the mesiodistal root angulation did not significantly alter between PAN-CBCT and CBCT images. While the angulation of the left first molar and the upper right lateral incisors was significantly different between PAN and CBCT images, the angulation of the top lateral incisors was shown to be significantly different between PAN and PAN-CBCT images. Interestingly, according to the findings of the cross-sectional study by Farhadian N, et al. [54], when compared to CBCT, the anterior teeth presented more parallelism in PAN and PAN-CBCT. In contrast, the posterior area did not differ between 2D radiography and CBCT. The authors concluded that when the head is positioned slightly downhill or ideally, the interdental angles in the anterior portion in PAN and PAN-CBCT images are closer to those in 3D CBCT. In this regard, it is necessary to conduct more research to ascertain the situations in which CBCT tests clearly outperform traditional 2D exams, hence supporting their application [50].

### 4.4. Limitations and Future Perspectives

The 3D imaging capabilities of CBCT enable orthodontists to detect, identify, and measure dental and skeletal structures and parameters with greater accuracy than conventional 2D radiography. This is a crucial consideration in orthodontic treatment, where minimizing adverse effects on tooth structure is paramount [76]. However, different limitations are associated with the use of CBCT. A common limitation in the existing literature on its use in orthodontics is the lack of standardized imaging protocols. Divergent settings for the exposure parameters and variations in patient positioning can introduce inconsistencies into the acquired data, potentially affecting the reproducibility and comparability of the findings across studies. Moreover, the cumulative effects of multiple CBCT scans, especially in the context of ongoing orthodontic treatment requiring repeated imaging, should be carefully evaluated to ensure the best clinical practice and patients’ safety [77]. Striking a balance between the diagnostic benefits and minimizing radiation risks is an ongoing challenge. The use of CBCT should be balanced, avoiding overdiagnosis and overtreatment [78].

One of the main limitations of the present scoping review is related to the design of the studies included. In fact, as reported in the Results section, most of the articles were cross-sectional studies, retrospective studies, or studies on cadavers’ skulls. Future research should prioritize longitudinal studies utilizing CBCT to track its impact on orthodontic diagnosis and interventions over time. Identifying the predictors of treatment outcomes based on CBCT findings may contribute to a more personalized and effective approach to orthodontic care [79]. Actually, 3D CBCT should be considered when any other low-dose radiographic modality cannot yield adequate diagnostic information for orthodontic diagnosis, treatment, and follow-up. For example, it could be indicated to evaluate the exact position of ectopic and/or impacted teeth associated with root resorption of the near teeth. In other cases, CBCT could be considered in cases of pathological processes such as odontomas and/or cysts complicating teeth eruption or for therapeutic planning and follow-up of selected complex malocclusions and/or syndromes. However, 3D evaluation should be balanced, taking into account its clinical necessity and justifiability and patients’ age and size since children and young adults are more susceptible to the effects of radiation exposure due to the higher sensitivity of their organs, as well as their longer expected life spans, resulting in a greater cumulative effect [74]. In this regard, newer CBCT models offer low-exposure alternatives to reduce the long-term risks of X-ray exposure in orthodontic populations. With these modern models, some scan protocols resulted in effective doses comparable with those in conventional PAN examinations, although significant dose reductions were accompanied by significant reductions in image quality. Dentists and radiologists should always adopt dose exposure reduction strategies when prescribing radiographic examinations.

Moreover, few studies have been included on other important topics in the orthodontic field, such as the accuracy of miniscrew positioning, determining cervical vertebrae maturity, and evaluating the accuracy of transverse jaw–base and dental relationship measurements. These limitations do not allow us to provide clinical indications on these applications. However, the present scoping review highlights the need to conduct further research, especially that with a longitudinal design.

Thanks to the introduction of cone beam technology, CBCT has emerged as a potential low-dose alternative to conventional CT in other healthcare areas beyond dentistry as well [80,81]. Actually, the use of radiographs is the clinician’s decision when a question cannot be answered clinically. Radiation exposure is directly related to the settings used and the areas exposed. Technological advances in both 2D and 3D radiography allow for a significant reduction in radiation exposure when they are used. Not all systems are the same, and using a now-obsolete 2D system might expose patients to more radiation than using a new 3D radiography system with pulse technology. Radiation exposure is part of the risk/benefit ratio decision that every clinician needs to make when deciding on their next step [68,82]. Future collaborative initiatives within the orthodontic community could contribute to the establishment of standardized imaging protocols for CBCT. A consensus on the best practices in terms of patient positioning, exposure parameters, and image analysis could enhance the reliability and comparability of research findings [83].

## 5. Conclusions

The choice between 3D CBCT or CBCT-generated radiography and conventional 2D radiography in orthodontics involves careful consideration of the specific clinical context, the complexity of the case, and the balance between its diagnostic advantages and associated limitations. While 3D imaging technologies offer unparalleled insights into the craniofacial complex, a judicious and case-specific approach to their integration into orthodontic practice is necessary. Tailoring the imaging approach to the specific clinical requirements of each patient remains essential to ensure the optimal diagnostic accuracy while minimizing risks. Future studies with a prospective design and standardized imaging protocols are encouraged to facilitate the development of a consensus on the best practices.

## Figures and Tables

**Table 1:** Inclusion and exclusion criteria.

Domain	Inclusion Criteria	Exclusion Criteria
Language	English	Other languages
Topic	3D CBCT vs. 2D radiography in orthodontics	Articles that did not compare 3D CBCT vs. 2D radiography in orthodontics
Study design	Cross-sectional, cohort, case–control, randomized and non-randomized controlled clinical trials, and retrospective studies	Opinion articles, theses, conference reports, case reports, case series, and any types of review articles
Availability	Full-text	Only a title and abstract

**Table 2:** Summary of the studies included regarding the use of CBCT for cephalometric analysis.

Study	Aim	Study Design	Sample	Main Results
Aksoy S, et al., 2016 [37]	To evaluate the accuracy of angular and linear measurements made in 3D CBCT-generated cephalograms and 2D lateral cephalometric images using different rendering tools.	Retrospective	15 individuals (10 F, 5 M)	It was discovered that while the 2D and 3D cephalograms produced by different rendering programs were comparable, 3D software has difficulty reproducing measurements on curved surfaces. Future efforts in this specific field should focus on designing new landmarks and analysis tools in tandem with the advancement of 3D imaging.
Cattaneo PM, et al., 2008 [25]	To evaluate the differences between cephalometric measures made on traditional cephalograms and those made on CBCT-synthesized images.	Cross-sectional	37 patients (20 F, 17 M)	There was no difference in the computed measures between the imaging approaches. Conventional headfilms can be effectively replaced by cephalograms generated using CBCT.
Chen MH, et al., 2014 [26]	To compare traditional digital cephalograms with CBCT-synthesized 2D lateral cephalograms in order to assess the intra-observer reliability of landmark recognition.	Cross-sectional	20 patients (13 F, 7 M)	The two cephalometric modalities produced landmark recognition errors ranging from 0.18 mm to 1.67 mm in both the horizontal and vertical directions. In CBCT-synthesized cephalograms, intra-observer errors of more than 1 mm were exhibited for fewer landmarks. The menton, lower central incisor edge, and lower central incisor root apex landmarks in the horizontal dimension and the pogonion, gnathion, menton, upper central incisor root apex, lower central incisor root apex, and lower molar landmarks in the vertical dimension showed significantly better reliability in the CBCT-synthesized cephalograms. For the identification of orthodontic problems, CBCT-synthesized lateral cephalograms can effectively replace traditional cephalograms.
Chien PC, et al., 2009 [27]	To evaluate the dependability of digital 2D lateral cephalograms and 3D CBCT patient images for landmark recognition.	Cross-sectional	10 lateral cephalograms and corresponding CBCT	When compared to 2D cephalograms, CBCT generally provided enhanced intra-observer and inter-observer reliability for certain landmarks in vivo.
Damstra J, et al., 2011 [32]	To use a three-dimensional analysis based on the midsagittal plane in order to compare cephalometric data in 2D vs. 3D.	Observational in dry skulls	10 skulls examined with conventional lateral cephalograms and CBCT	When the two- and three-dimensional measures were compared, it was evident that there were no significant differences (*p* = 0.41–1.00) and that the results were trustworthy (ICC > 0.88). Applying the stated midsagittal three-dimensional technique yielded data from the cephalometric studies that were interchangeable and comparable.
Hariharan A, et al., 2016 [28]	To compare digital 2D lateral cephalograms and CBCT images of the entire skull and half-skull in terms of the reliability of cephalometric measurements.	Cross-sectional	30 patients	Although further research is needed to confirm whether CBCT images of the entire skull can be utilized for cephalometrics, CBCT has the potential to be employed, particularly for half-skull images. In orthodontic diagnosis and treatment planning, 2D cephalometry is still the gold standard and is not readily superseded by 3D cephalometry.
Jung PK, et al., 2015 [38]	To examine whether it is possible to use a 2D standard in 3D analysis by comparing the angles and lengths measured from a midsagittal projection in 3D CBCT with those measured using 2D lateral cephalometric radiography (LCR).	Retrospective	50 patients (12 M, 38 F)	The 2D-LCR normative values could be employed in the practical 3D-CBCT analysis technique, which makes use of midsagittal projection. Reorientation caused some alterations in the measures, although they were not clinically significant.
Kumar V, et al., 2008 [29]	To compare measures from traditional cephalometric radiography with those from orthogonal and perspective projections used in CBCT-derived lateral cephalograms.	Cross-sectional	31 patients (13 M, 18 F)	The measurements derived from traditional radiography images are comparable to those derived from CBCT-produced cephalograms. For orthodontic diagnosis, CBCT scans may therefore typically be obtained without the need for further conventional imaging.
Ludlow JB, et al., 2009 [30]	To evaluate the accuracy of landmark recognition between traditional lateral cephalograms (Cephs) and CBCT.	Cross-sectional	20 presurgical orthodontic patients	The MPR presentations of CBCT offered higher-accuracy identification of conventional cephalometric landmarks. Condylion, gonion, and orbitale points were identified with more precision, overcoming the issue of these bilateral markers being superimposed in Cephs. Inadequate delineation of the landmarks in the third dimension is likely linked to the greater variability in some landmarks in the mediolateral direction.
Ogawa N, et al., 2010 [39]	To measure the magnification of cephalograms, evaluate the dimensional accuracy of CBCT images, develop a method for cephalometric analysis using CBCT images, and compare the cephalometric analytical values obtained from CBCT images with those from existing cephalograms in order to create a new cephalometric analysis method based on 3D data obtained using CBCT.	Retrospective	50 patients (16 M and 34 F)	The outcomes showed that the analytical approach made it easier to conduct a cephalometric analysis using CBCT, which, in turn, made it possible to compare CBCT images directly with cephalograms that already existed. Compared to traditional cephalograms, the range of inter-operator variability in the measurements produced through the cephalometric analysis employing CBCT was smaller.
Oz U, et al., 2011 [40]	To compare CBCT-generated cephalograms which are obtained from a 3D volumetric rendering tool with linear and angular measurements made in 2D traditional cephalometric images.	Retrospective	11 patients (6 F and 5 M)	It was discovered that the measurements from in vivo CBCT-generated cephalograms matched those from traditional images. Therefore, because CBCT exams involve more radiation, they should only be performed when the 3D data included can enhance the course of therapy.
Perrotti G, et al., 2023 [31]	To compare data from 2D “norms” for the facial measures and 3D measurements from direct anthropometry, as well as soft tissue measurements of the same distances acquired from 3D CBCT reconstructions with 2D cephalometric radiograms.	Cross-sectional	40 patients	Evaluations based on 2D and 3D measures revealed no statistically significant differences, except for the distances to the True Vertical Line in 2D (for both males and females) and the Labial superius prominence in females. All 3D measures deviated significantly from Arnett’s and anthropometric Farkas’ “norms”. For 70% of measurements, the average discrepancy between Farkas’ “norms” and 3D measures was less than 3 mm. The findings indicate that 3D soft tissue examination enables thorough diagnostic determination. Verification of the 3D “norms” requires a larger sample.
Pittayapat P, et al., 2014 [33]	To assess the precision of linear measures on three imaging modalities: 3D models using CBCT data, lateral cephalograms from a machine using a 3 m source-to-mid-sagittal-plane distance (SMD), and lateral cephalograms from a cephalometric machine with a 1.5 m SMD.	Observational in dry skulls	21 dry human skulls	Improved observer agreement was obtained using 3D measurements. Compared to a 3 m SMD cephalogram, the measurements based on CBCT and a 1.5 m SMD cephalogram were more accurate. When compared to 2D methods, these results showed the linear measurements’ precision and the dependability of 3D measurements based on CBCT data. Future research should concentrate on how 3D cephalometry is applied in clinical settings.
Van Vlijmen OJ, et al., 2009 [34]	To assess the comparability of measurements made in CBCT-constructed cephalometric radiography from human skulls to measurements made in traditional cephalometric radiography.	Observational in dry skulls	40 dry human skulls	For every measurement, there was good intra-observer reliability. When measurements were made in cephalometric radiography derived from CBCT scans, the repeatability was higher than that of measurements in traditional cephalometric radiography. There was no discernible variation in the measures between built and traditional cephalometric radiography that would be clinically significant.
Van Vlijmen OJ, et al., 2010 [35]	To assess the comparability of 3D measurements on human skull models made from CBCT data with measurements in traditional cephalometric radiography.	Observational in dry skulls	40 dry human skulls	When compared to measurements made in 3D models, the repeatability of measurements made in traditional cephalometric radiography was greater. A clinically significant disparity between measurements made in traditional cephalometric radiography and on 3D models was discovered for a select few parameters. Measurements made in 3D models of the same skull can differ dramatically from measurements made in traditional cephalometric radiography. When there are only 2D recordings from the past, the authors advise against using 3D tracings for longitudinal study.
Yang S, et al., 2014 [36]	To look into the consistency of linear measures between CBCT orthogonally generated cephalograms and conventional cephalograms and to assess the impact of various magnifications on these comparisons based on a simulation technique.	Observational in dry skulls	12 dry human skulls	Linear measurements in conventional cephalograms and orthogonally generated CBCT cephalograms were in agreement. Midsagittal magnification may be used to make up for differences between these two imaging modalities. In clinical practice, head orientation and landmark identification need to be closely observed. It also suggests that once CBCT data are obtained, further conventional cephalograms are not necessary. During this transitional time, the norms for orthogonally synthesized cephalograms from CBCT may be calculated using longitudinal data obtained from traditional cephalograms.

**Table 3:** Summary of the studies included regarding the use of CBCT for root resorption evaluation.

Study	Aim	Study Design	Sample	Main Results
Alamadi E, et al., 2017 [41]	To assess and compare the precision of 2D PA and PAN and 3D CBCT radiographic techniques in measuring slanted root resorptions in relation to the true resorptions as the histological gold standard.	Retrospective RCT study (extraction vs. non-extraction of a deciduous canine)	67 patients (40 M, 27 F)	PAN miscalculated the length of the roots on the sides with the least and most resorption. Small resorptions were more challenging to document and could only be reliably evaluated using CBCT. Using PA and PAN, the root resorption scores were underestimated. When it came to the evaluation of linear measurements, PAN was very different from CBCT and PA. When it comes to measuring and rating slanted root resorptions, CBCT is the most precise method available.
Alqerbaan A, et al., 2011 [43]	To evaluate the location of palatally displaced canines and the identification of canine-induced maxillary incisor root resorption using CBCT and PAN images.	Cross-sectional	60 patients (37 F and 23 M)	According to this study’s findings, CBCT is more sensitive than traditional radiography in locating canines and detecting neighboring teeth’s root resorption.
Alqerbaan A, et al., 2009 [44]	To assess the diagnostic accuracy of CBCT or PAN for the detection of simulated canine-induced external root resorption lesions in maxillary lateral incisors.	Observational study in dry skulls	A skull from a child cadaver in early mixed dentition. There were eight maxillary left lateral incisors removed with simulated root resorption cavities.	When it comes to identifying simulated external root resorption cavities, CBCT radiography is more sensitive than conventional radiography.
Michielsens H, et al., 2023 [42]	To evaluate how well the Malmgren index performs for determining root resorption (RR) in 2D panoramic radiograph sand CBCT.	Retrospective	20 patients (14 F, 6 M)	For 3D images, particularly axial ones, the original Malmgren index is inappropriate, as employing dichotomized values (resorption yes/no) causes RR to be overestimated. Early in orthodontic therapy, low-dose CBCT of the upper incisors might identify RR with good diagnostic accuracy, particularly in individuals who have experienced oral trauma or have a family history of RR.
Saccomanno S, et al., 2018 [45]	To confirm whether radiographic images may be useful in medical and legal scenarios, as well as confirming the accuracy of the radiographic image and the best radiological approaches to the diagnosis of root resorption in order to prevent, treat, and minimize it.	In vitro	PA and PAN performed on 19 extracted teeth	For the diagnosis of root resorption, PAN was not helpful. PA examination was the most reliable and impartial tool for identifying root resorption. The higher radiation dosage and cost associated with CBCT restrict its availability in most clinical settings, despite the literature’s suggestion that it is a trustworthy method for diagnosing root resorption problems.

PA: periapical radiography; PAN: panoramic radiography.

**Table 4:** Summary of the studies included regarding the use of CBCT for evaluation of root angulation or tooth position.

Study	Aim	Study Design	Sample	Main Results
Alquareer A, et al., 2021 [46]	To compare the clinical judgments made based on the interpretation of PAN vs. CBCT images for root angulation correction and root closeness.	Retrospective	36 radiographic patient records	PAN-based clinical decisions regarding root angulation had comparable statistical reliability and substantial agreement with CBCT-based clinical decisions.
Alqerban A, et al., 2014 [47]	To examine the benefits of employing CBCT in the orthodontic treatment of maxillary impacted canines, as well as the course of therapy.	Retrospective cohort	118 treated patients: CBCT group (58, 39 F/19 M) and conventional group (60, 31 F/29 M)	In instances where the maxillary canine impaction symptoms were more severe, CBCT was utilized. When CBCT was used, the diagnostic performance and success rates in more challenging instances were enhanced to a degree comparable to those in simpler cases handled with 2D data.
Alqerban A, et al., 2014 [51]	To evaluate the 3D data obtained from CBCT scans against the orthodontic treatment plans for impacted maxillary canines based on traditional orthodontic treatment records.	Prospective cohort	40 individuals (26 F and 14 M) with two sets of information: conventional records and CBCT	When using conventional and CBCT sets for treatment planning, there was no statistically significant difference. It has been demonstrated that, with a high degree of confidence, CBCT scans can provide valuable orthodontic treatment planning information that is comparable to that in conventional planning.
Barakaat AA, et al., 2023 [53]	CBCT, PAN, and panoramic images in CBCT (PAN-CBCT) were used as radiographic images to quantify the mesiodistal root angulation of the teeth.	Cross-sectional	22 patients (12 M, 10 F)	Mesiodistal root angulation was not significantly altered between PAN-CBCT and CBCT images. While the angulation of the left first molar and the upper right lateral incisors was significant between the PAN and CBCT images, the angulation of the top lateral incisors was shown to be significant between the PAN and PAN-CBCT images.
Botticelli S, et al., 2011 [52]	To determine whether patients with unerupted maxillary canines vary in any way from those who do not in terms of the diagnostic information obtained from 3D CBCT vs. traditional 2D radiography.	Prospective cohort	27 patients (17 F and 10 M)	The results showed that the two approaches did not localize the affected canines in the same way. This discrepancy can be attributed to various factors that affect conventional 2D radiography, including distortion, magnification, and superimposition of anatomical structures located in different planes of space. The diagnostic and treatment planning changed to take a more clinically oriented approach due to the enhanced assessment of the space conditions in the arch and the higher accuracy in the localization of the canines achieved with CBCT.
Bouwens DG, et al., 2011 [48]	To use CBCT scans and post-treatment PAN radiography images to compare mesiodistal root angulations.	Retrospective	35 orthognathic surgery patients	There was a significant statistical difference (*p* < 0.001) in the global test for both arches, suggesting that there was variation in the root angulation between the values obtained from the PAN and CBCT images. A comprehensive clinical evaluation of the dentition should support the cautious assessment of mesiodistal tooth angulation using PAN radiography.
Farhadian N, et al., 2014 [54]	Comparing CBCT to traditional panoramic and panoramic-like radiography in terms of the precision of measurements made using various head orientations between the long axis of neighboring teeth.	Cross-sectional	30 patients (12 M, 18 F)	When compared to CBCT, the anterior teeth presented more parallelism on panoramic imaging, whether conventional or panoramic-like. The posterior area, however, did not differ between radiography and CBCT. When the head is positioned slightly downhill or ideally, the interdental angles in the anterior portion of a panoramic-like picture are closer to those in the CBCT readings.
Nasseh I, et al., 2017 [49]	To analyze and compare the mesiodistal root angulations in PAN and PAN-CBCT.	Retrospective	40 patients (18 M, 22 F)	Since different approaches yield different results in different sections of arches, caution must be taken when using PAN-CBCT. If the volume is appropriately adjusted to provide a PAN-like image, the images may help evaluate mesiodistal root angulations.
Pico C, et al., 2017 [50]	To evaluate and draw conclusions on how the perception of upper canine impaction varied between seeing a set of CBCT reconstructions and a panoramic picture.	Retrospective	20 patients (10 M, 10 F)	Different information was obtained on tooth position (particularly with regard to the mesiodistal apex position and the labio-palatal cusp position), as well as the evaluation of root resorption, from the analyses of PAN vs. CBCT image reconstructions. It is necessary to conduct more research to ascertain the situations in which CBCT tests clearly outperform traditional 2D exams, hence supporting their application.
Wriedt S, et al., 2012 [55]	To determine whether 3D CBCT offers a better assessment of the location and likelihood of alignment of impacted upper canines compared to PAN.	Cross-over	21 patients	When the canine inclination in PAN exceeds 30°, when root resorption of the neighboring teeth is suspected, and/or when the canine apex is not clearly discernible, implying dilaceration of the canine root, small-volume CBCT may be justified as an addition to routine PAN.

PAN: panoramic radiography.

**Table 5:** Summary of the studies included regarding the use of CBCT for miscellaneous applications.

Study	Aim	Study Design	Sample	Main Results
Aboudara C, et al., 2009 [59]	To compare imaging data on the size of the nasopharyngeal airway from a 3D CBCT scan and a lateral cephalometric headfilm in teenage individuals.	Cross-sectional	35 adolescents (27 F, 8 M).	The relationship between airway area and volume was found to be reasonably high (r = 0.75); the greater the size, the higher the volume. In the lateral headfilms, however, there was a great deal of variation in the airway volumes of patients with comparatively identical airway surfaces in the cephalometric headfilms.
Benneman R, et al., 2012 [61]	To contrast the diagnostic capability of CBCT and PAN as common dental diagnostic procedures for the estimation of miniscrew positioning.	Observational study in dry skulls	9 macerated skulls in which one miniscrew was positioned per quadrant.	An approximate assessment of the miniscrew location with respect to the adjacent anatomic structures was made possible by the PAN. The probands’ evaluations were vague, nevertheless. Significant variations were seen in determining the screw’s closeness to the tooth root. Because of this, 3D diagnostics should be used in patients if there is any uncertainty, ideally before any surgical intervention.
Echevarría-Sánchez G, et al., 2020 [56]	To compare lateral cephalograms (LCs) with cephalograms produced from CBCT to estimate the cervical vertebrae maturity (CVM) in a population of Peruvians.	Retrospective	40 patients (18 M, 22 F)	As a substitute approach to evaluating CVM, CBCT is a trustworthy technique that may be applied. CBCT scans might be an important resource for orthodontists when it comes to techniques for estimating CVM.
Sears CR, et al., 2011 [58]	To use CBCT in order to examine the pharyngeal airway in 3D and assess whether the airway alterations before and after orthognathic surgery correspond with 2D lateral cephalograms and 3D CBCT images.	Prospective cohort	20 patients (13 F, 7 M)	An airway measurement technique that may be used for both 2D and 3D radiography was described. In individuals who had undergone orthognathic surgery, correlations between the linear and volumetric measures of the segmented airway were discovered; however, these relationships were often modest.
Tai B, et al., 2014 [60]	To evaluate the precision of traditionally recommended reference points for transverse jaw–base and dental relationship measurements using traditional Postero-Anterior Cephalometry (PAC) and CBCT.	Cross-sectional	31 patients (12 M, 19 F)	Clinicians are advised to exercise caution when evaluating and making judgments pertaining to measurements in the transverse dimensions obtained from PAC. When using standard clinical criteria to determine who needs maxillary expansion treatments, PAC is more likely to identify people incorrectly. Both PAC and CBCT procedures involve considerable mistakes related to the identification of structures that indicate the breadth of the mandible, which needs more research. The capacity of a clinician to recognize pertinent landmarks is thought to be significantly impacted by the confusing effects of underlying soft tissues.
Yilmaz H, et al., 2022 [57]	To evaluate the correctness of the root location when placing digital indirect brackets using CBCT and panoramic radiography.	Retrospective	27 patients (16 F, 11 M)	The angular deviation of the bracket’s position was clinically considerably affected by the use of panoramic radiography or CBCT in digital bonding. When placing brackets in digital indirect bonding, these results should be taken into account. Digital indirect bonding should not be the only justification for using CBCT in patients, even in the event of positive outcomes.

## Data Availability

Not applicable.
